# A validation of the first genome-wide association study of calcaneus ultrasound parameters in the European Male Ageing Study

**DOI:** 10.1186/1471-2350-12-19

**Published:** 2011-01-28

**Authors:** Delnaz Roshandel, Wendy Thomson, Stephen R Pye, Steven Boonen, Herman Borghs, Dirk Vanderschueren, Ilpo T Huhtaniemi, Judith E Adams, Kate A Ward, Gyorgy Bartfai, Felipe Casanueva, Joseph D Finn, Gianni Forti, Aleksander Giwercman, Thang S Han, Krzysztof Kula, Michael E Lean, Neil Pendleton, Margus Punab, Alan J Silman, Frederick C Wu, Kate L Holliday, Terence W O'Neill

**Affiliations:** 1 Arthritis Research UK Epidemiology Unit, The University of Manchester, Manchester Academic Health Science Centre, Manchester, UK; 2 Leuven University Division of Geriatric Medicine, Katholieke Universiteit Leuven, Leuven, Belgium; 3 Leuven University Center for Metabolic Bone Diseases, Katholieke Universiteit Leuven, Leuven, Belgium; 4 Department of Andrology and Endocrinology, Katholieke Universiteit Leuven, Leuven, Belgium; 5 Department of Surgery and Cancer, Imperial College London, Hammersmith Campus, London, UK; 6 Clinical Radiology, Manchester Royal Infirmary & Manchester Academic Health Science Centre, Manchester, UK; 7 MRC-Human Nutrition Research, Cambridge, UK; 8 Department of Obstetrics, Gynaecology and Andrology, Albert Szent-Gyorgy Medical University, Szeged, Hungary; 9 Department of Medicine, Santiago de Compostela University, Complejo Hospitalario Universitario de Santiago (CHUS), CIBER de Fisiopatologı'a Obesidad y Nutricion (CB06/03), Instituto Salud Carlos III, Santiago de Compostela, Spain; 10 Andrology Unit, Department of Clinical Physiopathology, University of Florence, Florence, Italy; 11 Scanian Andrology Centre, Department of Urology, Malmö University Hospital, University of Lund, Lund, Sweden; 12 Department of Human Nutrition, University of Glasgow, Glasgow, Scotland; 13 Department of Andrology and Reproductive Endocrinology, Medical University of Lodz, Lodz, Poland; 14 Clinical Gerontology, The University of Manchester, Manchester Academic Health Science Centre, Hope Hospital, Salford, UK; 15 Andrology Unit, United Laboratories of Tartu University Clinics, Tartu, Estonia; 16 Department of Endocrinology, Manchester Royal Infirmary, The University of Manchester, Manchester Academic Health Science Centre, Manchester, UK

## Abstract

**Background:**

A number of single nucleotide polymorphisms (SNPs) have been associated with broadband ultrasound attenuation (BUA) and speed of sound (SOS) as measured by quantitative ultrasound (QUS) at the calcaneus in the Framingham 100K genome-wide association study (GWAS) but have not been validated in independent studies. The aim of this analysis was to determine if these SNPs are associated with QUS measurements assessed in a large independent population of European middle-aged and elderly men. The association between these SNPs and bone mineral density (BMD) measured using dual-energy X-ray absorptiometry (DXA) was also tested.

**Methods:**

Men aged 40-79 years (N = 2960) were recruited from population registers in seven European centres for participation in an observational study of male ageing, the European Male Ageing Study (EMAS). QUS at the calcaneus was measured in all subjects and blood was taken for genetic analysis. Lumbar spine (LS), femoral neck (FN) and total hip (TH) BMD were measured by DXA in a subsample of 620 men in two centres. SNPs associated with BUA or SOS in the Framingham study with p < 10^-4 ^were selected and genotyped using SEQUENOM technology. Linear regression was used to test for the association between SNPs and standardised (SD) bone outcomes under an additive genetic model adjusting for centre. The same direction of effect and p < 0.05 indicated replication.

**Results:**

Thirty-four of 38 selected SNPs were successfully genotyped in 2377 men. Suggestive evidence of replication was observed for a single SNP, rs3754032, which was associated with a higher SOS (β(SD) = 0.07, p = 0.032) but not BUA (β(SD) = 0.02, p = 0.505) and is located in the 3'UTR of *WDR77 *(WD repeat domain 77) also known as androgen receptor cofactor p44. A single SNP, rs238358, was associated with BMD at the LS (β(SD) = -0.22, p = 0.014), FN (β(SD) = -0.31,p = 0.001) and TH (β(SD) = -0.36, p = 0.002) in a locus previously associated with LS BMD in large-scale GWAS, incorporating *AKAP11 *and *RANKL*.

**Conclusions:**

We found suggestive evidence of association between a single SNP located in the 3'UTR of *WDR77 *with calcaneal ultrasound parameters. The majority of SNPs, associated with QUS parameters in the Framingham Study, were not replicated in an independent population sample of European men.

## Background

Quantitative ultrasound (QUS) of the calcaneus is a widely used method for the assessment of bone health. Prospective studies confirm a positive relationship between a decline in ultrasound parameters (broad band ultrasound attenuation [BUA] and speed of sound [SOS]) and fracture risk [1-3].

Genetic factors are important determinants of calcaneus ultrasound parameters. Family and twin studies have shown that heritability of BUA and SOS at the calcaneus is 52-59% and 45-75%, respectively [4-7]. The proportion of population variation in ultrasound parameters explained by genetic factors is similar in men and women, though there is also evidence suggesting a gender-specific component to the overall genetic variance [8]. Genome-wide association studies (GWAS) are now widely used for identifying genetic associations with complex traits. During the past three years, a number of GWAS exploring various bone phenotypes have been published. The majority of these studies have focused on bone mineral density (BMD); however, a number of single nucleotide polymorphisms (SNPs) have also been reported in association with BUA and SOS in the Framingham 100K GWAS, the first published GWAS of bone phenotypes [9]. These findings remain to be validated in independent population samples.

Our study aimed to validate the most significant SNP associations with BUA and SOS from the Framingham 100K GWAS utilizing an independent population of middle-aged and elderly men recruited in the European Male Ageing Study (EMAS). We also investigated, in a subgroup of men, the association between these SNPs and BMD as assessed using dual energy x-ray absorptiometry (DXA).

## Methods

### Study Participants

Men aged 40-79 years were recruited into EMAS from population registers in 8 European centres (Manchester, UK; Leuven, Belgium; Tartu, Estonia; Lodz, Poland; Szeged, Hungary; Florence, Italy; Santiago de Compostela, Spain; Malmö, Sweden). Subjects were invited to participate by letter of invitation and those who agreed were invited to attend for a more comprehensive assessment including a blood sample for genetic analysis and QUS at the calcaneus. A subsample of subjects in two centres (Manchester and Leuven) had BMD measured by DXA at the lumbar spine (LS), femoral neck (FN) and total hip (TH). Participants were excluded from the analysis if they reported that they themselves or one of their parents or grandparents was born outside Europe or North America, or if they reported use of anti-osteoporotic medications or systemic glucocorticoids. Ethical approval in each centre was obtained in accordance with local practice and requirements, and subjects gave informed consent; approval for the genetic analysis described here was obtained for seven of the eight centres (all centres except for Malmö, Sweden). Analysis was restricted therefore to subjects from these seven centres.

### Anthropometric and Lifestyle Measurements

Weight to the nearest 10 g and height to the nearest mm were measured using calibrated scales stadiometer. Body mass index (BMI) was calculated as weight divided by height squared (kg/m^2^). Cigarette smoking was assessed by standard questionnaire. Physical activity was measured using the physical activity scale for the elderly (PASE) [10].

### Bone Assessments

#### QUS

Heel ultrasound measurements were made at the left calcaneus, using the Sahara Clinical Sonometer (Hologic, Bedford, Massachusetts, USA), in all centres following a standardized protocol. Each centre used the same machine model, which was calibrated daily with the physical phantom provided by the manufacturer. Outputs included broadband ultrasound attenuation (BUA) (dB/MHz) and speed of sound (SOS) (m/s). Quality control (QC) was performed in each centre following the instructions of the manufacturer. All QC results were compiled and checked for stability throughout the study in Leuven. To ascertain the short-term precision of the method in this population, duplicate measurements were performed in 20 randomly selected subjects in Leuven. The in vivo coefficient of variation (CV) was 2.8% and 0.3% for BUA and SOS, respectively. Repeat measurements (N = 10) were performed on a roving phantom at each of the eight centres. Standardized CVs for within machine variability ranged by centre from 1.0% to 5.6% for SOS and from 0.7% to 2.7% for BUA. Standardized CVs for between machine variability were 4.8% and 9.7% for BUA and SOS, respectively.

#### DXA

Bone densitometry scans were carried out in the Manchester and Leuven subsets of EMAS. Both sites used DXA QDR 4500A devices of the same manufacturer (Hologic, Inc, Waltham, MA, USA). BMD (g/cm^2^) was measured at the LS (L1 to L4) and proximal femur (FN and TH). All scans and measurements were performed by trained and experienced DXA technicians. The Hologic Spine Phantom was scanned daily to monitor the device performance and long-term stability. The precision of these measurements in the LS, FN and TH were 0.57%, 1.28% and 0.56% in Leuven, and 0.97%, 1.29% and 0.97% in Manchester, respectively. Both devices were cross-calibrated with the European Spine Phantom [11].

### Genotyping and Quality Control

#### DNA Extraction

DNA was extracted from leucocytes in venous blood samples using standard phenol-chloroform extraction and stored at -80°C prior to further analysis.

#### SNPs Selection

The data from the Framingham 100K GWAS were extracted from the database of Genotypes and Phenotypes (dbGaP) (http://www.ncbi.nlm.nih.gov/projects/gap/cgi-bin/study.cgi?id=phs000007) in January 2008. Stringent quality control was applied to the data; call rate ≥ 95% and Hardy-Weinberg equilibrium (HWE) p > 10^-4^. Subsequently, SNPs with minor allele frequency (MAF) ≥5% associated with BUA or SOS (p < 10^-4^) in the 100K Framingham GWAS based on multivariable adjusted (age, height, BMI, smoking, physical activity, estrogen therapy) additive generalized estimating equation (GEE) model were selected for genotyping.

For the gene (±10 Kb flanks) showing evidence of replication in EMAS, additional pair-wise tag SNPs (r^2^≥0.8, MAF ≥5%) were selected using HapMap CEPH SNP data (http://www.hapmap.org) and Tagger implemented in Haploview 4.0 [12].

#### Genotyping and Quality Control

SEQUENOM MassARRAY technology was used for genotyping all SNPs following the manufacturer's instructions (http://www.sequenom.com). Sample and assay quality control thresholds were set to 90%. Allele frequencies were tested for deviation from HWE in the total population and the SNPs with p ≤ 0.05 were excluded from analysis. STATA (9.2) was used for calculating allele frequencies and quality control.

### Statistical Analysis

The outcome variables (BUA, SOS and BMD at the LS, FN and TH) were standardised (z-scores). The association between the SNPs and the standardised outcome variables was tested using linear regression under an additive genetic model with adjustments made for centre. Multivariable analysis was also conducted to further adjust for age, BMI, height, smoking (both current and ever) and physical activity in keeping with the Framingham Study. All analyses were performed using PLINK (Version 1.07) [13]. Results are presented as mean change in outcome (β coefficient) with 95% confidence intervals (95%CI) for each copy of the minor allele. The criteria for replication were that a SNP needs to be associated with ultrasound measures with p < 0.05 with the effect estimate (β coefficient) in the same direction as reported in the Framingham study. The interaction between SNP and centre was tested for the SNPs associated with the outcome variables to test for between centre heterogeneity using STATA (9.2).

The statistical power was calculated using Quanto v1.2.3 software [14].

## Results

### Subject Characteristics

Of the 2960 men recruited from the seven centres, 2653 consented to participate in genetic analysis, 215 of which were excluded due to failing sample quality control (n = 101), reporting at least one of their parents or grandparents being born outside Europe or North America (n = 17) or reporting use of anti-osteoporotic medications or systemic glucocorticoids (n = 97). In total, 2438 men, mean (±SD) age 60 (±11) years old, were included in the analysis of which 2377 had QUS performed. BMD analysis was performed in a subset of 620 subjects. Mean values for BMD and QUS parameters are presented in Table 1. BUA and SOS were highly correlated (r^2 ^= 0.81, p < 0.001).

**Table 1:** Subject Characteristics; DXA and QUS parameters

	Florence	Leuven	Lodz	Manchester	Santiago	Szeged	Tartu	All Centres
**QUS**	**N = 398**	**N = 328**	**N = 369**	**N = 337**	**N = 336**	**N = 348**	**N = 261**	**N = 2377**
**BUA (dB/MHz)**	77.05 (17.71)	82.43 (17.63)	79.90 (18.28)	87.62 (17.46)	84.00 (21.34)	71.09 (18.92)	79.30 (17.75)	80.09 (19.12)
**SOS (m/s)**	1542.32 (32.97)	1561.91 (31.66)	1547.46 (32.66)	1560.54 (34.51)	1557.86 (39.74)	1543.42 (30.09)	1543.54 (32.94)	1550.89 (34.53)
**DXA BMD (g/cm**^**2**^**)**	-	**N = 328**	-	**N = 292**	-	-	-	**N = 620**
**Lumbar spine**	-	1.04 (0.17)	-	1.07 (0.19)	-	-	-	1.06 (0.18)
**Femoral neck**	-	0.80 (0.12)	-	0.82 (0.14)	-	-	-	0.81 (0.13)
**Total hip**	-	1.01 (0.14)	-	1.02 (0.15)	-	-	-	1.01 (0.14)

Data are shown in mean (standard deviation). QUS: Quantitative ultrasound, BUA: Broadband ultrasound attenuation, SOS: Speed of sound, DXA: Dual energy X-ray absorptiometry, BMD: Bone mineral density

### Power of Study

For BUA (mean ± SD = 80.1 ± 19.1 dB/MHz) and SOS (mean ± SD = 1550.9 ± 34.5 m/s); with 5% type I error, MAFs of 0.05-0.45, and 2377 individuals, there was 80% power to detect differences of greater than 0.2 SD for MAF = 0.05 and 0.1 SD for MAF = 0.45 under an additive genetic model. For BMD at the LS (mean ± SD = 1.06 ± 0.18 g/cm^2^), FN (mean ± SD = 0.81 ± 0.13 g/cm^2^) and TH (mean ± SD = 1.01 ± 0.14 g/cm^2^); with 5% type I error, MAFs of 0.05-0.45, and 620 individuals, there was 80% power to detect differences of greater than 0.4 SD for MAF = 0.05 and 0.2 SD for MAF = 0.45 under an additive genetic model.

### Genotyping

Thirty-eight SNPs associated with BUA (19 SNPs) and/or SOS (26 SNPs) in the Framingham study with p ≤ 10^-4 ^were selected. All SNPs associated with BUA with p ≤ 10^-4 ^were also associated with SOS with p < 0.05, and vice versa. The details of the selected SNPs are shown in Additional file 1: Supplementary Table S1. Four SNPs (rs10513725, rs1936473, rs2108167 and rs4954265) failed genotyping. All remaining 34 SNPs were successfully genotyped and passed quality control.

In addition, 4 SNPs (rs1891756, rs1264913, rs12040764 and rs3754032) which tag a gene, *WDR77*, showing evidence of replication, and its 10 Kb flanking region were selected for genotyping. One of these SNPs, rs12040764, failed genotyping; the others were successfully genotyped and passed quality control. The successfully genotyped SNPs gave 80% coverage of the SNPs with a MAF of more than 5% in *WDR77 *and its 10 kb flanking region (chr1, 111774036-111803353).

### Genetic Association Analysis

#### QUS

**Figure 1:**
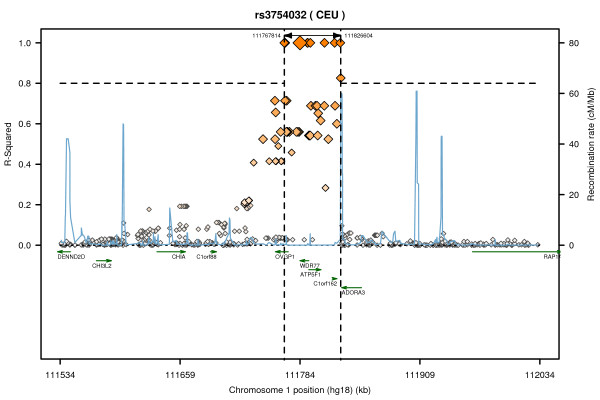
**Regional LD plot for rs3754032**. The plot shows the linkage disequilibrium between rs3754032 and HapMap SNPs within 250 Kb, recombination rate and the genes in the region using Annotation and Proxy Search (SNAP) (http://www.broadinstitute.org/mpg/snap/index.php). The two dashed vertical lines show the location of HapMap SNPs which are in high LD (r^2 ^≥ 0.8) with rs3754032.

The results for BUA and SOS are shown in Table 2. None of the SNPs were associated with BUA in EMAS whereas a single SNP, rs3754032, was associated with SOS. This SNP, which was associated with a higher SOS (β(SD) (95% CI) =0.07 (0.01, 0.13), p = 0.032) in our study, was associated with higher levels of both BUA (p = 8.75 × 10^-5^) and SOS (p = 0.01) in the Framingham study. In our study, no significant association was observed between rs3754032 and BUA (β(SD) (95%CI) =0.02 (-0.04, 0.08), p = 0.505). In order to account for the number of independent SNPs (r^2^<0.8) tested (N = 31), SNP associations would need to reach a p-value of <0.0016 to achieve statistical significance therefore the association between rs3754032 and SOS should be considered suggestive of replication. The regional linkage disequilibrium (LD) plot for rs3754032 is shown in Figure 1.

**Table 2:** Characteristics of the SNPs and their association with BUA and SOS

						Adjusted for centre				Adjusted for centre, age, BMI, height, smoking and PASE score			
						BUA		SOS		BUA		SOS	
Chr	Position	SNP	Alleles	MAF (%)	Nearest Gene within 500 kb	β (SD)	p	β (SD)	p	β (SD)	p	β (SD)	p
1	55553409	rs1807871	T > C	13.1	USP24	0.00	0.949	0.00	0.988	-0.02	0.645	0.00	0.949
1	92085352	rs2799516	G > A	9.0	TGFBR3	0.04	0.455	0.05	0.347	0.02	0.694	0.04	0.386
1	92099224	rs2046737	C > T	8.5	TGFBR3	0.06	0.231	0.06	0.240	0.05	0.339	0.06	0.267
1	111784127	rs3754032	T > A	26.0	WDR77	0.02	0.505	0.07	0.032	0.01	0.665	0.06	0.085
2	70027133	rs10496176	T > C	15.4	MXD1	0.03	0.440	0.01	0.874	0.02	0.619	0.00	0.950
2	82913481	rs10496276	T > G	19.2		0.02	0.638	0.01	0.696	0.00	0.944	0.00	0.940
2	118250857	rs1433527	C > A	45.3	DDX18	-0.04	0.110	-0.04	0.211	-0.05	0.081	-0.03	0.262
2	135824309	rs10496734	G > A	14.9	ZRANB3	-0.04	0.363	-0.03	0.447	-0.06	0.172	-0.06	0.139
2	158955976	rs2251471	T > C	6.7	CCDC148	0.09	0.123	0.12	0.027	0.10	0.060	0.14	0.012
3	30344099	rs1587126	A > C	33.7	TGFBR2	0.01	0.769	-0.01	0.665	-0.01	0.856	-0.02	0.496
3	99203733	rs1492053	G > A	37.0	GABRR3	0.00	0.963	0.00	0.909	0.01	0.704	0.01	0.674
3	163243925	rs10513577	T > C	31.0		0.00	0.991	0.01	0.773	0.02	0.559	0.03	0.320
3	163273126	rs1033059	A > G	31.0		0.00	0.943	0.01	0.815	0.02	0.588	0.03	0.339
3	163310228	rs951937	T > A	25.6		-0.02	0.568	0.00	0.975	-0.01	0.873	0.01	0.665
4	9933258	rs9291683	G > A	46.8	ZNF518B	0.04	0.171	0.04	0.168	0.03	0.334	0.03	0.245
4	57967285	rs10517393	G > T	22.8	IGFBP7	0.01	0.795	-0.03	0.341	0.02	0.481	-0.02	0.639
4	132163115	rs2055391	C > T	8.4		-0.01	0.920	0.00	0.996	0.01	0.789	0.01	0.800
4	182246126	rs7659755	A > G	18.3		-0.02	0.534	-0.04	0.225	-0.01	0.889	-0.03	0.366
5	54231548	rs2099082	T > C	23.6	ESM1	0.00	0.903	-0.01	0.767	0.01	0.853	0.00	0.957
5	157166116	rs10515754	T > C	6.0	CLINT1	0.06	0.293	0.05	0.422	0.10	0.086	0.10	0.082
6	91568115	rs9294466	A > T	15.0	MAP3K7	0.04	0.301	0.05	0.211	0.05	0.172	0.06	0.129
6	96483564	rs6925466	C > T	38.9	FUT9	0.00	0.870	0.00	0.888	-0.02	0.503	-0.02	0.493
7	14495103	rs7786503	A > C	8.0	DGKB	0.02	0.643	0.02	0.759	0.02	0.672	0.02	0.722
7	14497256	rs10499444	C > G	7.7	DGKB	0.01	0.869	-0.01	0.898	0.01	0.884	0.00	0.942
7	30896348	rs6462230	G > C	10.7	FLJ22374	0.04	0.396	0.06	0.221	0.02	0.732	0.03	0.485
7	147333625	rs2214681	G > A	42.5	CNTNAP2	0.00	0.950	-0.01	0.723	-0.01	0.798	-0.02	0.476
10	59991493	rs1649053	T > C	38.8	BICC1	-0.02	0.583	-0.01	0.829	-0.04	0.151	-0.03	0.324
10	127169837	rs10510144	G > A	28.9	MMP21	0.05	0.127	0.06	0.048	0.05	0.083	0.08	0.016
13	20860773	rs1409071	G > A	41.9	ZDHHC20	0.05	0.106	0.06	0.047	0.02	0.559	0.03	0.288
13	41736674	rs238358	G > A	9.5	AKAP11	0.03	0.536	0.03	0.516	-0.01	0.916	-0.01	0.896
13	92478066	rs10492621	G > A	24.2	GPC5	-0.06	0.068	-0.07	0.042	-0.07	0.035	-0.07	0.045
14	86696500	rs10513893	C > T	7.2		0.01	0.830	-0.01	0.821	0.04	0.449	0.03	0.551
16	26362052	rs8049649	A > T	17.6	HS3ST4	0.06	0.080	0.05	0.197	0.06	0.103	0.05	0.191
20	23097739	rs10485640	A > G	9.7	CD93	-0.05	0.274	-0.05	0.334	-0.05	0.273	-0.06	0.187

Chr: Chromosome, MAF: Minor allele frequency, BUA: Broadband ultrasound attenuation, SOS: Speed of sound, PASE: Physical activity scale for the elderly; β(SD), effect estimates are shown as standardized values (standard deviations) for each copy of the minor allele.

After further adjustment for age, BMI, height, smoking and physical activity; although the estimated effect of rs3754032 on SOS did not significantly alter, the p value was slightly increased (β(SD) (95%CI) =0.06 (-0.01, 0.12), p = 0.085).

The SNP rs3754032 is located in *WDR77*, therefore tag SNPs for *WDR77 *and its 10 Kb flanking regions were subsequently genotyped. However, no significant associations between the tag SNPs in *WDR77 *(rs1891756 and rs1264913) and ultrasound measures were observed.

#### DXA

**Figure 2:**
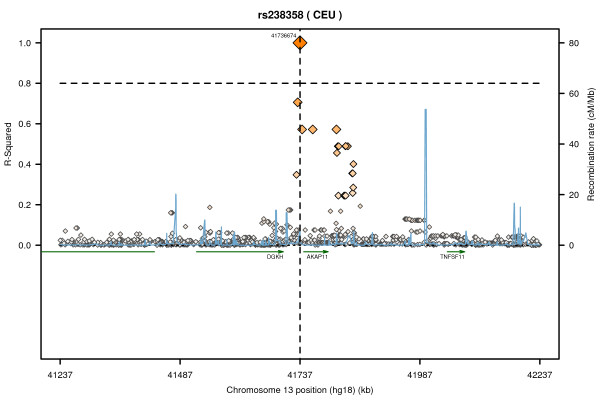
**Regional LD plot for rs238358**. The plot shows the linkage disequilibrium between rs238358 and the other HapMap SNPs in 500 kb, recombination rate and the genes in the region using Annotation and Proxy Search (SNAP) (http://www.broadinstitute.org/mpg/snap/index.php). The two dashed vertical lines show the location of HapMap SNPs which are in high LD (r^2 ^≥ 0.8) with rs238358.

A single SNP, rs238358, on chromosome 13 was associated with BMD at LS (β(SD) (95%CI) =-0.22 (-0.44, -0.06), p = 0.014), FN (β(SD) (95%CI) =-0.31 (-0.54, -0.15), p = 0.001) and TH (β(SD) (95%CI) =-0.36 (-0.50, -0.14), p = 0.002). However, this SNP was not associated with BMD in the Framingham study. After further adjustment for age, BMI, height, smoking and physical activity; rs238358 was still suggestively associated with BMD at LS (β(SD) (95%CI) =-0.22 (-0.39, -0.06), p = 0.025), FN (β(SD) (95%CI) =-0.31 (-0.46, -0.08), p = 0.004) and TH (β(SD) (95%CI) =-0.29 (-0.43, -0.07), p = 0.003). The regional LD plot for rs238358 is shown in Figure 2.

## Discussion

In this study we attempted to validate findings from the Framingham 100K GWAS study by testing the association of the most significantly associated SNPs (p < 1 × 10^-4^), after applying stringent quality control criteria, with BUA and SOS ultrasound measures at the calcaneus in an independent sample of 2377 unrelated European men. Evidence of replication was observed for a single SNP, rs3754032.

This SNP which was highly associated with BUA and modestly associated with SOS in the Framingham study was only associated with SOS in EMAS. The association, however, does not remain significant if corrected for multiple testing and should be considered suggestive of replication.

The SNP rs3754032 is located on chromosome 1, in the 3'UTR of *WDR77 *(WD repeat domain 77) also known as androgen receptor cofactor p44. The androgen receptor activates transcription of different target genes in response to androgens [15,16], which have anabolic effects on male bone metabolism. Acute onset of androgen deficiency such as surgically induced hypogonadism or pharmacological androgen deprivation therapy causes rapid bone loss and increases risk of fracture in men [17]. Androgen receptor cofactor p44 is one of a number of cofactors which increases androgen receptor transcriptional activities in the nucleus. It also acts as a splicing associated factor in the cytoplasm [15,16].

Four other genes are also located in this region of chromosome 1 including *ADORA3 *(adenosine A3 receptor), *OVGP1 *(oviductal glycoprotein 1), *ATP5F1 *(ATP synthase B chain) and *CHIA *(chitinase, acidic); and SNPs in this region are in moderate LD with rs3754032 (Figure 1). Adenosine A3 receptor is a cell surface receptor that mediates part of the anti-inflammatory effects of adenosine [18]. Interestingly, studies in rat suggest that adenosine A3 receptor agonists can preserve bone mass in adjuvant induced arthritis [18] and prevent bone destruction in osteoarthritis [19]. We did not find any evidence in the literature linking the other three genes (*OVGP1*, *ATP5F1 *and *CHIA*) and bone metabolism. Whilst *WDR77 *represents a good candidate, we found no association between other SNPs within this gene and ultrasound parameters suggesting a single independent effect. Further work is required to validate this association in a large cohort and to determine the causal effect in this region.

The majority of the SNP associations with BUA and SOS in the Framingham study were not replicated in our population. Lack of replication could be due to a number of factors. First of all, the two populations differed in geographical location and gender; the Framingham study (Massachusetts, USA) included 1141 subjects with 57% women whereas our population included 2377 men from seven countries across Europe. In addition, using Bonferroni correction for the number of SNPs tested in the Framingham study (N = 70,987), a p-value < 7 × 10^-7 ^would be required to reach genome-wide significance level. However, none of the SNPs reached this level possibly because of small sample size. We set our cut off less stringently at p < 10^-4 ^so that we would not miss genuine associations. Therefore, there was a greater chance that the selected SNPs were false positives due to multiple testing. In addition, QUS is a less precise method than DXA and shows higher within-subject variability. There was also some evidence of between-centre variability in the QUS parameters, which may have reduced the likelihood of detecting true associations in EMAS.

Spurious results might have been produced in our data due to factors such as population stratification. We attempted to minimise population stratification by excluding subjects of non-European ancestry and we did not observed any heterogeneity of effect (by SNP centre interaction). However, the country of origin was assigned based on the subjects self-report and we were unable to explore population substructure using methods such as genomic control or principal component analysis as these require data on a large number of SNPs. Therefore, we cannot exclude the possibility of population stratification in our data.

Although the SNPs were selected based on their association with BUA and SOS, some of them were also associated with BMD (p < 0.05) in the Framingham study. In a sub-sample of our population (620 subjects) for whom DXA measures were available, a single SNP, rs238358, was associated with BMD at all three skeletal sites (LS, FN and TH). The association between rs238358 and FN BMD remained significant (p = 0.0009) even after applying a Bonferroni correction for the number of independent SNPs (r^2^<0.8) (p < 0.0016 (0.05/31)). This SNP is located on chromosome 13, about 8 Kb upstream of *AKAP11 *(A kinase anchor protein 11) and 35 Kb downstream of *DGKH *(diacylglycerol kinase, eta) (Figure 2). This SNP was not associated with BMD in the Framingham study but other SNPs in *AKAP11 *have previously been associated with LS BMD in a large-scale meta-analysis of GWAS at the genome-wide significant level [20]. We did not find any evidence in the literature connecting *DGKH *and bone metabolism.

The SNP rs238358 is also located about 300 Kb upstream of *TNFSF11 *(tumor necrosis factor superfamily, member 11) which encodes RANKL. *TNFSF11 *is located beyond a recombination hot spot and only modest LD (r^2^<0.2) exists between SNPs within this gene and rs238358. Therefore, rs238358 is unlikely to be a marker for a causal SNP within the gene. However, the upstream region in which rs238358 is located may contain elements influencing *TNFSF11 *regulation. RANKL is a member of RANKL/RANK/OPG signalling pathway which has an important role in bone remodelling and has been associated with BMD at the genome-wide significant level in GWAS [20-22] and in the EMAS population previously [23].

The SNPs included in the Affymetrix 100K array used in the Framingham GWAS have very limited tagging properties thus extensive areas of the genome were not adequately evaluated in the GWAS. Using genome-wide SNP chips with greater genome coverage in a large consortium which combines data from multiple cohorts will enable ascertainment of susceptibility loci for bone quality as assessed by QUS parameters, similar to that which has been carried out for DXA BMD at osteoporotic sites [20].

## Conclusions

We observed suggestive evidence of association between a single SNP located in the 3'UTR of *WDR77 *with the calcaneal ultrasound parameter, SOS. This association requires further validation in other independent populations. If this association is confirmed, fine mapping and functional studies will be needed to identify the causal variant. However, the majority of the SNPs associations identified in the recent Framingham GWAS of bone ultrasound phenotypes were not replicated in EMAS, an independent population sample of men.

## Competing interests

The authors declare that they have no competing interests.

## Authors' contributions

DR contributed to the design of the genetic study, performed the genotyping, conducted the analysis, contributed to the interpretation of the results and drafted the manuscript. WT and KLH conceived and contributed to the design of the genetic study and were involved in overseeing the analysis, contributing to the interpretation of the results and in the preparation of the final manuscript. SRP contributed to the interpretation of the results and preparation of the final manuscript. SB, HB, DV, ITH, JEA, KAW, GB, FC, JDF, GF, AG, KK, MP, AJS conceived and designed the European Male Ageing Study, acquired the subjects and data, and critically reviewed the manuscript. TSH, MEL, NP critically reviewed the manuscript. FCW led the European Male Ageing Study concept and design, acquired the subjects and data, and contributed to the interpretation of the results and the preparation of the final manuscript. TWO conceived and designed the study, acquired the subjects and data, oversaw the analysis, contributed to the interpretation of the results and preparation of the final manuscript. All authors read and approved the final manuscript.

## Pre-publication history

The pre-publication history for this paper can be accessed here:

http://www.biomedcentral.com/1471-2350/12/19/prepub

## Supplementary Material

Additional file 1 Supplementary Table S1: Genetic association results from the Framingham 100K GWAS for SNPs selected for genotyping Click here for file
