# Supplementary material for: A validation of the first genome-wide association study of calcaneus ultrasound parameters in the European Male Ageing Study
Source: BMC Med Genet. 2011 Jan 28;12:19. doi: 10.1186/1471-2350-12-19 (PMC3042372; doi:10.1186/1471-2350-12-19)
Supplement: Additional file 1 — Supplementary Table S1: Genetic association results from the Framingham 100K GWAS for SNPs selected for genotyping [file 1471-2350-12-19-S1.DOC]

**Supplementary Table 1**: Genetic association results from the Framingham 100K GWAS for SNPs selected for genotyping

|  |  |  |  |  |  | **BUA** | | **SOS** | |  |
| --- | --- | --- | --- | --- | --- | --- | --- | --- | --- | --- |
| **Chr** | **Position** | **SNP** | **Alleles** | **MA** | **MAF** | **GEE β (SD)^a^** | **p** | **GEE β (SD)^a^** | **p** | **Nearest Gene within 500 kb** |
| 1 | 55553409 | rs1807871 | AG | G | 0.13 | -0.2 | 1.08E-03 | -0.24 | 6.03E-05 | USP24 |
| 1 | 92085352 | rs2799516 | CT | T | 0.08 | -0.32 | 3.19E-05 | -0.24 | 8.87E-04 | TGFBR3 |
| 1 | 92099224 | rs2046737 | AG | A | 0.07 | 0.32 | 4.18E-05 | 0.28 | 7.82E-05 | TGFBR3 |
| 1 | 111784127 | rs3754032 | AT | T | 0.25 | 0.2 | 8.75E-05 | 0.12 | 0.01 | WDR77 |
| 2 | 70027133 | rs10496176 | CT | C | 0.18 | 0.21 | 8.59E-05 | 0.16 | 2.10E-03 | MXD1 |
| 2 | 82913481 | rs10496276 | GT | G | 0.17 | 0.19 | 1.42E-03 | 0.23 | 9.66E-05 |  |
| 2 | 118250857 | rs1433527 | AC | A | 0.48 | 0.15 | 2.00E-04 | 0.18 | 1.94E-05 | DDX18 |
| 2 | 135824309 | rs10496734 | AG | A | 0.11 | 0.24 | 3.77E-04 | 0.24 | 9.90E-05 | ZRANB3 |
| 2 | 136040695 | rs4954265 | AG | G | 0.10 | -0.3 | 2.00E-05 | -0.27 | 1.40E-05 | R3HDM1 |
| 2 | 158955976 | rs2251471 | AG | G | 0.05 | -0.37 | 1.55E-06 | -0.23 | 0.01 | CCDC148 |
| 3 | 30344099 | rs1587126 | GT | G | 0.35 | -0.14 | 3.66E-03 | -0.18 | 8.78E-05 | TGFBR2 |
| 3 | 99203733 | rs1492053 | CT | T | 0.39 | 0.17 | 9.45E-05 | 0.13 | 4.34E-03 | GABRR3 |
| 3 | 163243925 | rs10513577 | CT | C | 0.25 | 0.15 | 2.29E-03 | 0.18 | 4.06E-05 |  |
| 3 | 163273126 | rs1033059 | AG | G | 0.25 | -0.15 | 2.06E-03 | -0.18 | 6.22E-05 |  |
| 3 | 163310228 | rs951937 | AT | T | 0.19 | -0.18 | 1.47E-03 | -0.21 | 4.96E-05 |  |
| 3 | 176068402 | rs10513725 | CG | C | 0.11 | 0.26 | 3.97E-05 | 0.22 | 3.03E-04 | NAALADL2 |
| 4 | 9933258 | rs9291683 | CT | C | 0.50 | 0.2 | 2.07E-06 | 0.17 | 3.77E-05 | ZNF518B |
| 4 | 57967285 | rs10517393 | AC | A | 0.23 | -0.17 | 1.48E-03 | -0.22 | 3.32E-05 | IGFBP7 |
| 4 | 132163115 | rs2055391 | AG | A | 0.07 | 0.21 | 0.01 | 0.3 | 6.03E-05 |  |
| 4 | 182246126 | rs7659755 | CT | C | 0.18 | 0.21 | 7.58E-05 | 0.18 | 5.82E-04 |  |
| 5 | 54231548 | rs2099082 | CT | C | 0.23 | 0.16 | 2.03E-03 | 0.18 | 8.31E-05 | ESM1 |
| 5 | 157166116 | rs10515754 | CT | C | 0.06 | -0.41 | 4.19E-06 | -0.38 | 1.70E-05 | CLINT1 |
| 6 | 91568115 | rs9294466 | AT | T | 0.15 | -0.23 | 7.69E-05 | -0.2 | 2.14E-04 | MAP3K7 |
| 6 | 96483564 | rs6925466 | AG | A | 0.38 | 0.2 | 3.68E-05 | 0.19 | 3.00E-05 | FUT9 |
| 7 | 14495103 | rs7786503 | AC | C | 0.06 | -0.28 | 8.27E-04 | -0.3 | 4.04E-05 | DGKB |
| 7 | 14497256 | rs10499444 | CG | C | 0.06 | 0.25 | 2.09E-03 | 0.29 | 6.01E-05 | DGKB |
| 7 | 30896348 | rs6462230 | CG | G | 0.10 | -0.23 | 1.32E-03 | -0.28 | 1.23E-05 | FLJ22374 |
| 7 | 41684632 | rs2108167 | AC | A | 0.27 | 0.2 | 9.91E-05 | 0.12 | 0.02 | INHBA |
| 7 | 147333625 | rs2214681 | AG | A | 0.46 | -0.19 | 2.72E-06 | -0.17 | 4.54E-05 | CNTNAP2 |
| 9 | 90504661 | rs1936473 | AG | G | 0.41 | 0.13 | 3.43E-03 | 0.16 | 8.88E-05 | NXNL2 |
| 10 | 59991493 | rs1649053 | AG | G | 0.41 | -0.18 | 4.08E-05 | -0.19 | 1.52E-05 | BICC1 |
| 10 | 127169837 | rs10510144 | CT | T | 0.29 | -0.17 | 9.80E-05 | -0.12 | 6.58E-03 | MMP21 |
| 13 | 20860773 | rs1409071 | CT | T | 0.43 | -0.11 | 0.01 | -0.16 | 7.67E-05 | ZDHHC20 |
| 13 | 41736674 | rs238358 | AG | A | 0.07 | 0.19 | 0.02 | 0.27 | 6.09E-05 | AKAP11 |
| 13 | 92478066 | rs10492621 | AG | A | 0.20 | 0.18 | 1.33E-03 | 0.2 | 4.75E-05 | GPC5 |
| 14 | 86696500 | rs10513893 | AG | A | 0.09 | -0.27 | 6.21E-05 | -0.14 | 0.03 |  |
| 16 | 26362052 | rs8049649 | AT | T | 0.17 | -0.15 | 8.39E-03 | -0.21 | 6.72E-05 | HS3ST4 |
| 20 | 23097739 | rs10485640 | AG | G | 0.09 | -0.28 | 6.88E-05 | -0.21 | 3.06E-03 | CD93 |

Chr: Chromosome, MA: Minor allele, MAF: Minor allele frequency, BUA: Broadband ultrasound attenuation, SOS: Speed of sound, GEE: Generalized estimating equation, effect estimates are shown as standardized values (standard deviations) for each copy of the minor allele adjusted for ^a^ age, height, BMI, smoking, physical activity and oestrogen therapy
